# Uncovering proteome variations and concomitant quality changes of different drying methods *Cordyceps sinensis* by 4D-DIA structural proteomics

**DOI:** 10.3389/fnut.2025.1463780

**Published:** 2025-02-05

**Authors:** Mengjun Xiao, Chuyu Tang, Tao Wang, Min He, Yuling Li, Xiuzhang Li

**Affiliations:** State Key Laboratory of Plateau Ecology and Agriculture, Qinghai Academy of Animal and Veterinary Sciences, Qinghai University, Xining, China

**Keywords:** *Cordyceps sinensis*, drying method, 4D-DIA proteomics, quality, structural changes

## Abstract

**Introduction:**

*Cordyceps sinensis* is a fungus, serves dual purposes as both a medicinal herb and a food source. Due to its high water content, fresh *Cordyceps sinensis* is difficult to preserve, necessitating the drying necessary to process *Cordyceps sinensis*.

**Methods:**

Using 4D-DIA proteomics, researchers analyzed the proteome profiles of fresh *Cordyceps sinensis* (CK) under three different drying conditions: vacuum freeze-drying (FD), oven-drying (OD), and air-drying (AD). In addition, it was found that the protein and free sulfhydryl content of *Cordyceps sinensis* decreased significantly and the disulfide bond content increased after different drying methods.

**Results and discussion:**

A total of 3762 proteins were identified, showing variations between groups and high protein content. In the control groups consisting of fresh *Cordyceps sinensis* samples and the three drying methods, FD. vs CK exhibited the fewest differentially abundant proteins, with the majority being upregulated. On the other hand, CK vs OD displayed the greatest amount of distinct proteins, with a significant rise in both up-regulated and down-regulated proteins. Analysis of KEGG indicated that the distinct proteins were predominantly concentrated in pathways like the ribosome, synthesis of coenzymes, and metabolism of amino sugar and nucleotide sugar. Notably, there was a significant overlap between ribosome and ribosome biogenesis in eukaryotes pathways. The process of drying *Cordyceps sinensis* resulted in a significant upregulation of the expression of proteins linked to various metabolic pathways. This observation suggests that the drying treatment might activate or enhance certain biochemical processes within the organism, potentially influencing its overall metabolic activity. This finding highlights the importance of post-harvest dry methods on the biochemical properties of *Cordyceps sinensis*, which could have implications for its nutritional and medicinal value.This study provides a theoretical basis for the realization of *Cordyceps sinensis* resource utilization and storage methods, and provides theoretical support for guaranteeing the sustainable development of *Cordyceps sinensis* resources.

## 1 Introduction

*Cordyceps sinensis* (*C. sinensis*) is a fungus, boasts a nutrient content that exceeds that of ginseng. It can be used for medicinal purposes or consumed as a delicacy (1, 2). The wild *C. sinensis* is primarily found in high-altitude regions above 3,000 m. It contains sterols, polysaccharides, proteins, and other chemical components and nutrients that exhibit a more effective impact on the body’s functional recovery and disease defense (3, 4). Protein, a key active ingredient in natural *C. sinensis*. It is a rich compound abundant in minerals, vitamins, and essential amino acids, characterized by a unique structure and rich nutritional value (5, 6).

Recent research on *C. sinensis* proteins has increasingly delved into their immune-enhancing and anti-tumor properties, as well as their potential as a key active ingredient in the quality identification of *C. sinensis* (2, 7, 8). However, due to the high water content in fresh *C. sinensis*, up to 70%, the active ingredients are prone to deterioration and require a complex dehydration process during storage and transportation (9). Therefore, it is of great significance to study different drying methods to improve the *C. sinensis* quality of clover products.

Currently, vacuum freeze drying (FD), oven drying (OD), and air drying (AD) are the primary drying methods employed in the *C. sinensis* industry, each with its own advantages and disadvantages. AD is the most cost-effective option with simple technology that is not constrained by equipment, but it is susceptible to environmental factors. The OD method is known for its low equipment investment and ease of use. In conclusion, FD methods have advantages in terms of efficiency and energy saving, but they require high equipment and can be prioritized for the production of high-quality products, whereas OD and AD are low-tech but of variable quality. However, it is also associated with reduced energy efficiency and lower quality output when compared to other technologies (10, 11). Although vacuum freeze drying (FD) is considered to be a method that highly preserves the original quality of the material, it is costly and requires high equipment (12). A study utilizing high-performance liquid chromatography (HPLC) examined the impact of sun- and freeze-drying on the six main nucleosides and nucleobases of *C. sinensis*, providing a foundation for upcoming investigations. This study provides insights into evaluating drying methods and maintaining quality control (13). Another study examined the impact of hot air drying (HAD), vacuum freeze drying (FD), vacuum drying (VD), and microwave alternate hot air drying (MW-HAD) on the aromatic qualities and scent of *Cordyceps militaris*. The results showed that MW-HAD was an effective drying method to promote umami taste, and VFD could superiorly preserve volatiles and characteristic aroma compounds in dried *Cordyceps militaris* (14).

4D DIA proteomics, as an emerging technology tool, provides a comprehensive, high-throughput and high-sensitivity analysis platform for biopharmaceutical research. Through the steps of sample pretreatment, protein separation, mass spectrometry and data analysis, quantitative and differential analysis of proteins can be realized (15, 16). Prior research has investigated the proteomic variances in *C. sinensis*, comparing wild and cultivated strains through label-free mass spectrometry for quantitative analysis. Results indicated similarities in protein and metabolite makeup between naturally occurring and intentionally grown fungi, with some distinctions observed in caterpillar bodies versus stromata (8). Another study use a proteomic methodology to comprehensively uncover the protective impacts of *C. sinensis* against diethylnitrosamine-induced liver cancer in rats, along with its underlying mechanisms. The findings demonstrated that *C. sinensis* could mitigate liver cancer by influencing redox balance, protein ubiquitination processes, and transcription factors linked with tumor development (8, 17). However, there is limited literature on the application of 4D-DIA proteomics methods to compare the protein profiles of *C. sinensis* under varying drying techniques.

This study investigated the proteome of *C. sinensis* under various drying conditions, including vacuum freeze drying (FD), oven drying (OD), and air drying (AD), using cutting-edge 4D-DIA proteomics technology. The protein structure of *C. sinensis* was characterized and thoroughly investigated. This research addresses the gap in understanding the proteomics of *C. sinensis* under various drying methods and sheds light on the response mechanism of proteins in the deterioration of *C. sinensis* quality. The research results provide novel perspectives and a theoretical foundation for improving the technology used in preserving *C. sinensis*, and can also be a useful resource for consumers looking to make informed decisions when buying the product.

## 2 Materials and methods

### 2.1 Sample collection

Baohuitang Biotechnological Co. Ltd. in Xining City, Qinghai Province, China, provided 20 g of fresh *C. sinensis* (CK) samples collected in June 2023 from Maqin in Guoluo Tibetan Autonomous Prefecture. The samples were promptly delivered to the lab and stored at −80°C for future use. Four groups of *C. sinensis* were established, with three biological replicates in each group. The samples underwent three different drying techniques: air drying (AD), oven drying (OD), and vacuum-freeze drying (FD).

In the FD method, Fresh *C. sinensis* was evenly distributed across trays and subjected to freeze-drying for 3 h at −35°C in a vacuum freeze-dryer. Pre-cooling was necessary, followed by a 25-h freeze-drying process at a high vacuum and low temperature to ensure consistent weight. In contrast, *C. sinensis* was laid out on a tray and dried at 60°C in an electrically heated constant-temperature drying oven using the OD method. Samples were exposed to hot air until a stable weight was achieved before removal. Lastly, the AD method involved laying *C. sinensis* flat in a cool, dry environment with controlled ambient temperature (20–25°C) and relative humidity (50–65%) until a constant weight was attained.

### 2.2 Determination of protein, free sulfhydryl, and disulfide bond content

The study delved into the characteristics of proteins from fresh and dried *C. sinensis*, focusing on protein content, free sulfhydryl groups, and disulfide bonds as key indicators. The analysis kits utilized were acquired from Suzhou Keming Biotechnology Co., with testing protocols adhering to the guidelines outlined in the kits and associated literature (18–20).

### 2.3 Proteomics analysis

#### 2.3.1 Protein extraction and digestion

Protein extraction was carried out following the method described previously (7, 21). Appropriate amount of sample was removed in frozen state, transferred to MP shaking tube, and shaking was performed using a high-throughput tissue grinder for 3 times, each time for 40 S. Subsequently, the supernatant was taken by centrifugation at 4°C 12,000 g for 20 min, and an equal volume of Tris-saturated phenol was added, and shaken at 4°C vortex for 10 min; 4°C 12,000q centrifugation for 20 min was performed to take the phenol phase, and an equal volume of BPP solution was added, and shaken at 4C vortex for 10 min; and 4°C 12,000 g centrifugation for 20 min to take the phenol phase, add an equal volume of BPP solution, 4C vortex shaking for 10 min; 4°C 12,000q centrifugation for 20 min to take the phenol phase, add 5 times the volume of pre-cooled ammonium acetate methanol solution, −20°C overnight precipitation of proteins; the next day, 4°C 12,000 g centrifugation for 20 min to discard the supernatant, to the precipitate added 90% pre-cooled. The supernatant was discarded by centrifugation at 12,000 g for 20 min at 4°C the next day, 90% pre-cooled acetone was added to the precipitate and the supernatant was discarded by centrifugation, and the procedure was repeated twice. To summarize, proteins were isolated with a lysis buffer comprising 8 M urea, 1% SDS, and a mixture of protease inhibitors. Samples were sonicated above ice for 2 min at 20 KHZ. This was followed by centrifugation at 12,000 g for 20 min at 4°C to collect the protein supernatant. The protein concentration was assessed utilizing the BCA method. Subsequently, 15 μg of protein was loaded onto SDS-PAGE for electrophoretic analysis. The resulting electropherograms were utilized to evaluate the reproducibility within the group.

Each protein solution was sampled at 100 μg and mixed with Tri-ethylammonium bicarbonate buffer (TEAB). Tri (2-carboxyethyl) phosphine (TCEP) was then introduced to reach a final concentration of 10 mM, allowing the reaction to take place for 60 min at 37°C. Iodoacetamide (IAM) was included to achieve a final concentration of 40 mM, and the reaction continued for 40 min at 10,000 g. After centrifuging for 20 min, the resulting precipitate was obtained. Following this, the sample was dissolved in 100 μL of 100 mM TEAB, and trypsin was added at a ratio of 1:50 enzyme-to-protein for digestion overnight at 37°C. After trypsin digestion, an aliquot of the sample was vacuum centrifuged in a concentrator to evacuate the peptides. The peptides from trypsin digestion were dissolved in 0.1% TFA (trifluoroacetic acid), purified, and eluted with HLB. Peptide quantification was performed with the Peptide Quantification Kit from Thermo Fisher Scientific.

#### 2.3.2 LC–MS/MS analysis

The LC–MS/MS analysis utilized the EASY-nLC 1,200 chromatography instrument (Thermo Fisher Scientific, United States) in conjunction with the timsTOF Pro2 mass spectrometer (Bruker, Germany). Peptides were dissolved in the sample buffer for mass spectrometry and additional 10× iRT peptides were mixed in for DIA analysis. Data acquisition was carried out with the Compass HyStar software (Bruker, Germany). The analytical column employed was an Ionopticks UPLC Column, C18 1.6 μm with dimensions 250 mm x 75 μm, sourced from Ionopticks, United States. Chromatographic separation occurred over a period of 44 min using solvents: A (2% acetonitrile +0.1% formic acid) and B (80% acetonitrile +0.1% formic acid), at a flow rate of 300 nL/min. The gradient elution schedule was as follows: starting at 0 min with 3% B, increasing to 28% B at 33 min, then 44% B at 37 min, rising to 90% B by 40 min, and ending at 44 min. Post-separation via ACQUITY UPLC I-Class PLUS, samples were subjected to mass spectrometry analysis through DIA using a timsTOF Pro2 spectrometer from Bruker. The mode of ion detection was positive, with an ion source voltage of 1.5 kV. Both MS and MSMS datasets were collected and analyzed via TOF.

#### 2.3.3 Protein identification and quantitation

The raw data from DIA was processed through extraction of daughter ion peaks, correlation of iRT for retention time, selection of 6 peptides for each protein, and 3 daughter ions for each peptide for quantitative analysis. The criteria involved ensuring that the protein FDR was ≥0.01, peptide FDR was ≤0.01, peptide Confidence was ≥99%, XIC width was ≤75 ppm, and calculation of peak areas was conducted. Quantitative outcomes were achieved by summing the peak areas, followed by normalization through the total peak area ratio for differential screening of proteins and statistical analysis.

### 2.4 Data analysis

Proteins and their sequences were identified through mass spectrometry and then compared with various databases such as KEGG and databases related to subcellular localization. The screening criteria for differentially expressed proteins (DEPs) in *C. sinensis* samples were FC > 2 or FC < 0.5 and *p* < 0.05, and hierarchical clustering heatmap analysis (HCA), GO, and KEGG annotation enrichment analyses were performed for the differential proteins in *C. sinensis* samples from the four groups involved in this study. Differential protein interaction (PPI) analysis was performed using the STRING (Version 11.5) Protein Interaction Database,^1^ and a network diagram was constructed to obtain the interaction relationships between proteins.

## 3 Results and analysis

### 3.1 Determination of protein, sulfhydryl, and disulfide bonds content

Proteins, the building blocks of cells, are crucial for the survival of organisms and for various biological activities and interactions (22, 23). Among amino acid side-chain groups, sulfhydryl is the most reactive in protein degradation (24, 25). Protein oxidation levels are typically evaluated through the detection of free sulfhydryl and disulfide bonds (26, 27). Consequently, the protein content, disulfide bond, and free sulfhydryl group in different drying methods play a significant role in understanding protein structure and function.

**Figure 1:**
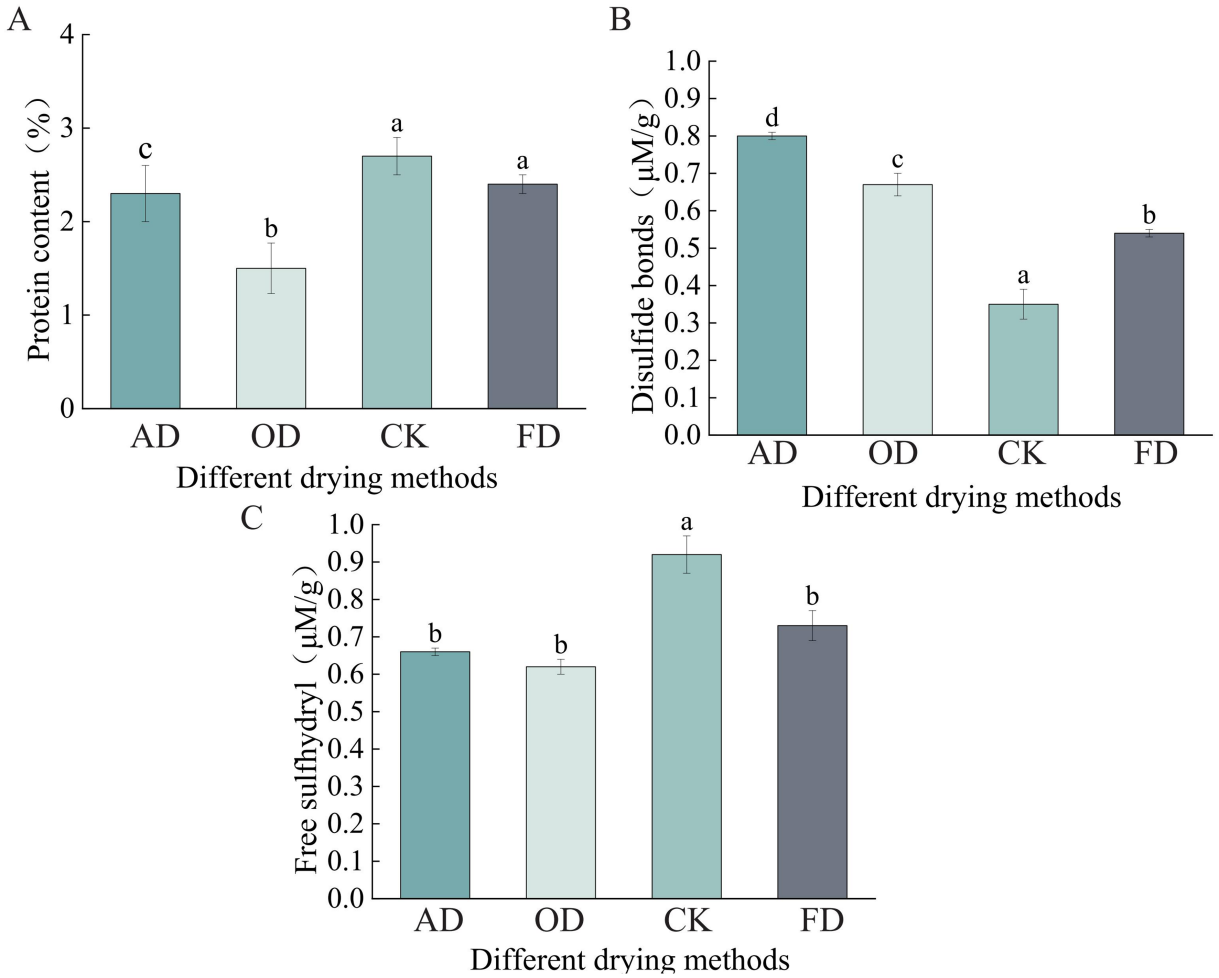
Impact of various drying techniques on *Cordyceps sinensis* structural characteristics. **(A)** Protein content; **(B)** disulfide bonds; **(C)** free sulfhydryl. Significant differences are represented by different lowercase letters.

There was no notable distinction in protein levels between the CK and FD samples (*p* > 0.05). A notable disparity in protein content was detected among the CK, AD, and OD samples (*p* < 0.05), with the protein content being the lowest in the OD sample (Figure 1A). In the analysis of disulfide bond content, a notable variation was observed across the four sample groups (*p* < 0.05), with the CK samples exhibiting the least amount of disulfide bonds (Figure 1B). The CK group exhibited a notable variance (*p* < 0.05) in free sulfhydryl content compared to the FD, OD, and AD groups, although there was no significant difference (*p* > 0.05) among the FD, OD, and AD groups (Figure 1C).

### 3.2 Protein identification and quantitation

**Figure 2:**
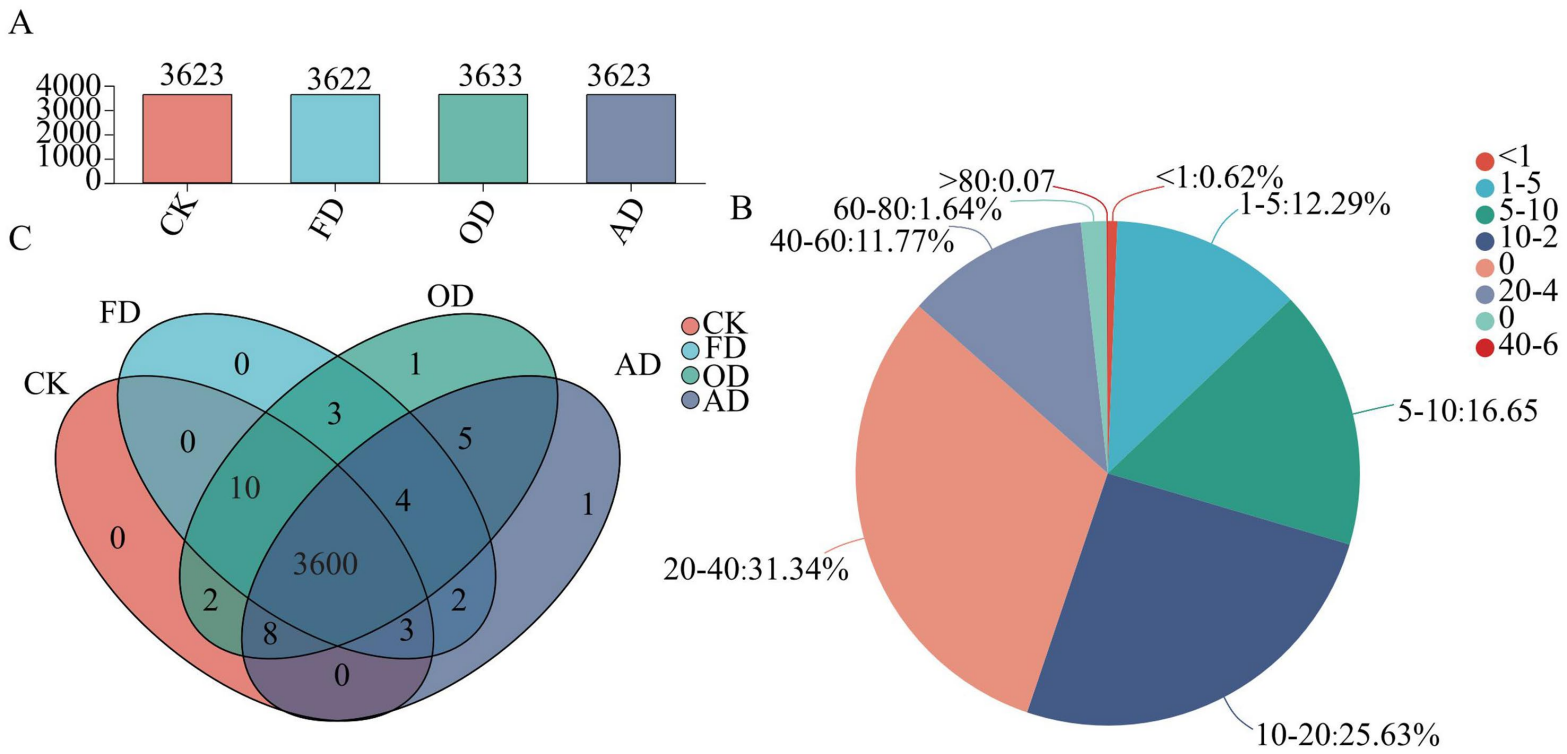
Panel **(A)** shows the distribution of identified proteins in both fresh and differently dried *C. sinensis* samples as represented by a column diagram; **(B)** the sequence coverage percentages of proteins assessed by identified peptides; **(C)** Venn diagram illustrating the distribution of identified proteins in both fresh and differently dried *C. sinensis* samples.

A quantitative proteomics analysis using 4D-DIA was conducted to evaluate protein expression levels and understand proteome changes in *C. sinensis* resulting from different drying methods. The study identified 8,377 distinct protein groups and 27,977 distinct peptides. Specifically, 3,623, 3,622, 3,633, and 3,623 proteins were identified in *C. sinensis* samples that were fresh, and dehydrated by FD, OD, and AD, respectively (Figure 2A). A sequence coverage greater than 10% was observed in over 70.44% of the proteins (see Figure 2B), suggesting the trustworthiness of the proteomic investigation. Examination of the Venn diagram for the proteins identified in each *C. sinensis* group demonstrated that the majority of the identified proteins (3600) were common to both fresh and different methods of drying *C. sinensis* (Figure 2C). Certain proteins in *C. sinensis* may undergo denaturation at high temperatures but remain relatively stable under the FD drying method. This could be attributed to the absence of microbial growth and enzyme activity in vacuum freeze-drying, allowing for better preservation of the original characteristics (28). Melanic reactions that occur during the drying process play a crucial role in food processing and storage. Maillard Reaction Products (MRPs) are formed as a result of these reactions, and they can alter the flavor and bioactivity of food by influencing protein expression levels (29–31).

### 3.3 Principal components analysis (PCA) and hierarchical cluster analysis (HCA)

**Figure 3:**
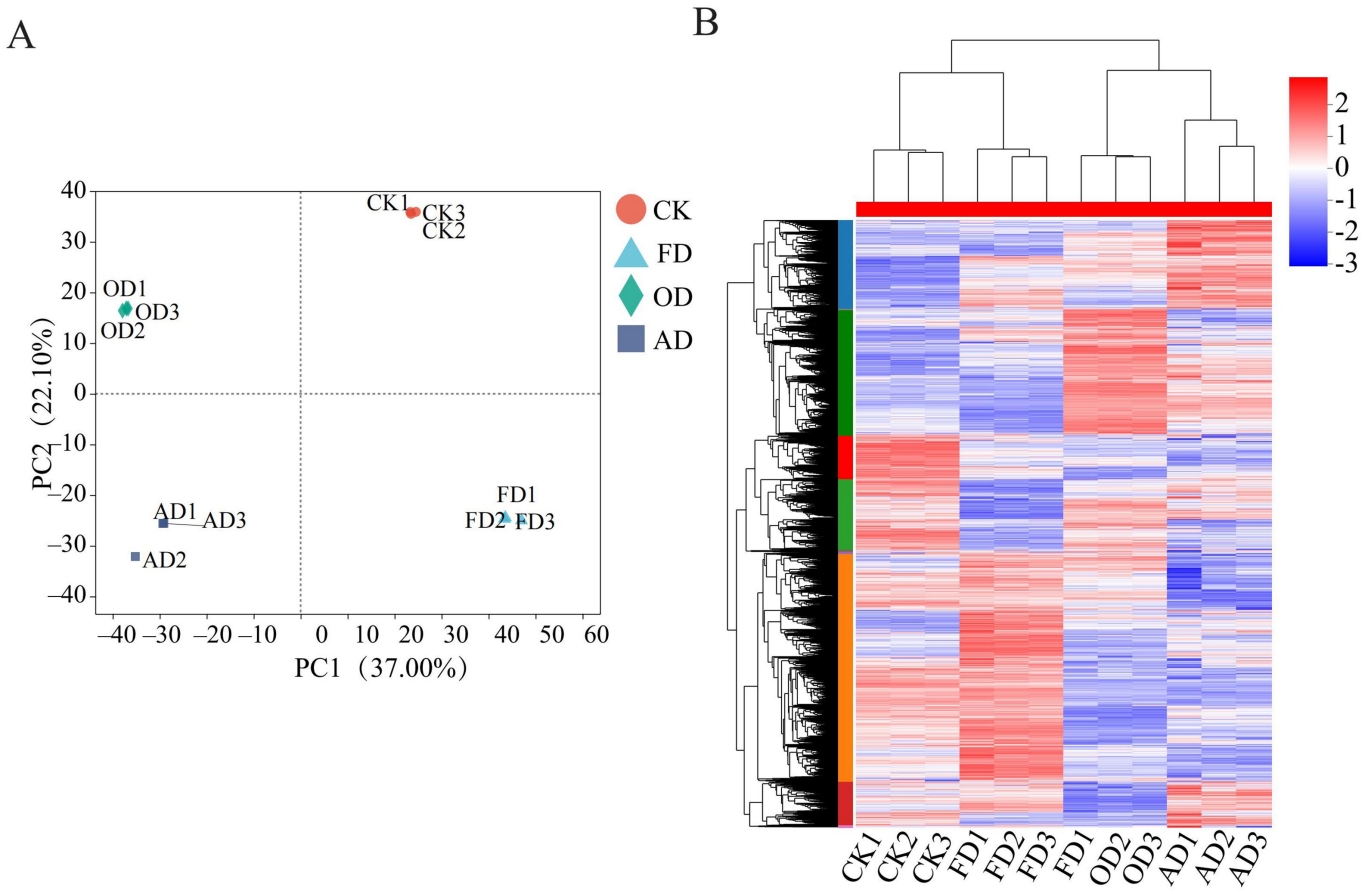
**(A)** Score plot of PCA. **(B)** Hierarchical quantitative protein clustering analysis of *C. sinensis* using different drying methods.

Principal component analysis (PCA), a method for unsupervised pattern recognition, was employed to visually represent the overall clustering patterns among various groups and the extent of variation among samples within each group (32). PCA assessment revealed that the samples could be distinctly categorized into four separate clusters relying on PC1, which accounted for 37.0%, and PC2, contributing 22.1% (Figure 3A). This distinction underscores the disparities in protein profiles among fresh samples and those subjected to diverse drying methods. The 4D-DIA approach’s reproducibility and dependability were attested by the tight aggregation of three biological replicates from every one of the four categories.

Further analysis of the quantified proteins revealed that the *C. sinensis* samples studied formed two main clusters (Figure 3B). Specifically, the OD and AD samples clustered together, while the CK and FD samples formed another distinct cluster. The hierarchical clustering analysis indicated significant differences in protein content between fresh *C. sinensis* and *C. sinensis* subjected to different drying methods. Interestingly, the protein content differences between *C. sinensis* dried using AD and OD methods were not pronounced, while the CK and FD groups exhibited a high similarity in protein content. These clustering patterns were largely consistent with the findings of the PCA.

### 3.4 Analysis of differentially abundant proteins (DEPs)

#### 3.4.1 DEPs and correlations

**Figure 4:**
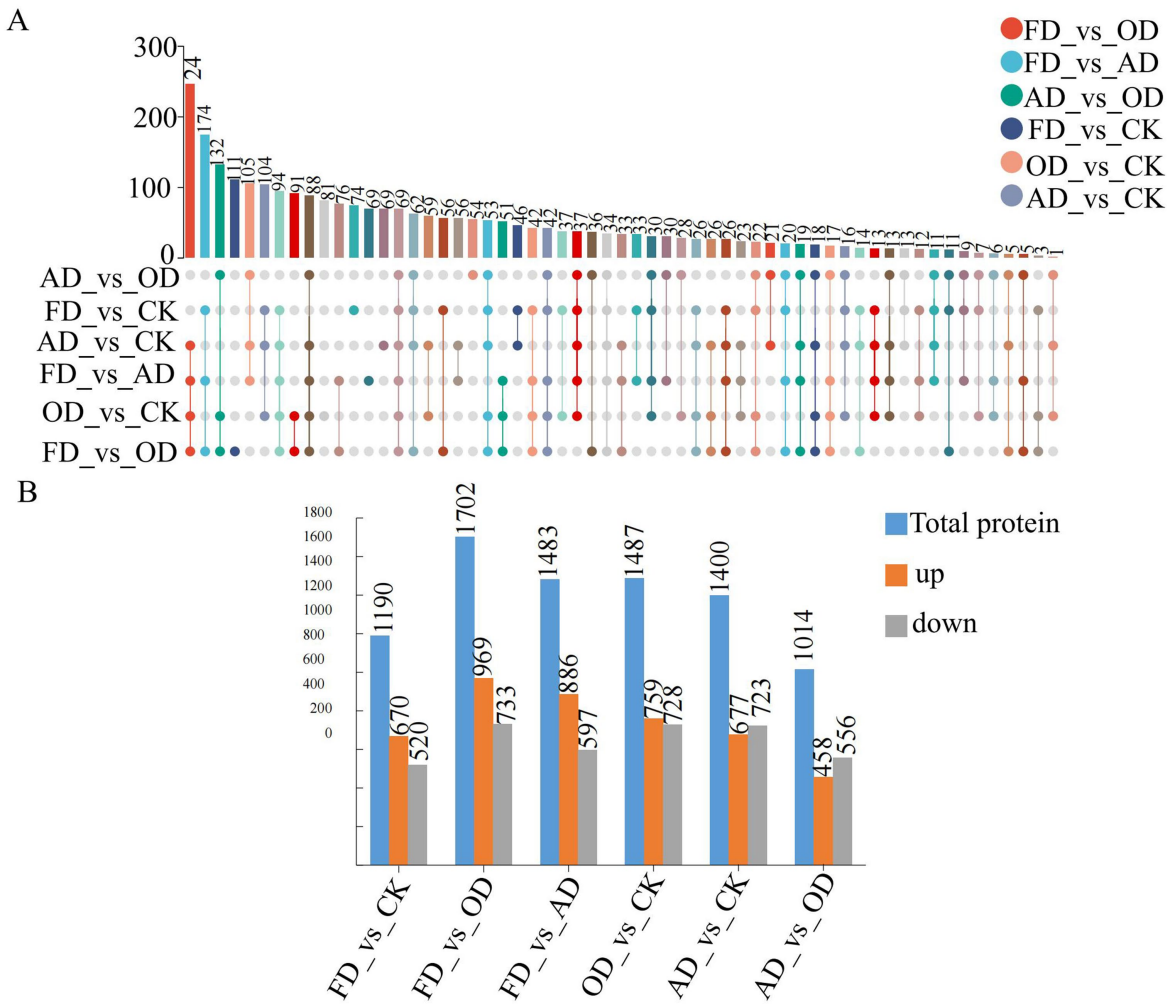
**(A)** Upset Venn diagram (the top bar chart is a count of the number of elements in different sets after taking the intersection, the individual dots below indicate elements specific to a grouping, and the lines between the dots indicate intersections specific to different groupings), **(B)** bar graphs (blue reflects the total number of differentially abundant proteins, orange denotes the quantity of up-regulated proteins, and gray indicates the number of down-regulated proteins).

In this study, proteins were classified as differentially abundant proteins (DEPs) if their fold change (FC) exceeded 2 or fell below 0.5, in addition to having a *p*-value less than 0.05 (Supplementary Table S1). Upset Venn diagrams and graphs were utilized to visually represent the significant differences in sample data across different groups. The upset Venn diagrams (Figure 4A) illustrated that 69 DEPs were proteins shared among all three groups. Furthermore, there were 37, 46, and 22 shared DEPs between FD vs. CK and OD vs. CK, FD vs. CK and AD vs. CK, and OD vs. CK and AD vs. CK, respectively. Additionally, there were 74, 81, and 69 exclusive DEPs in FD vs. CK, OD vs. CK, and AD vs. CK, respectively.

In the investigation, a total of 2,759 metabolites showing differential expression were uncovered. Out of these, 670 metabolites exhibited up-regulation, while 520 showed down-regulation when comparing FD to CK. Similarly, in the OD versus CK comparison, 759 metabolites were found to be upregulated and 728 down-regulated, whereas, between AD and CK, 677 metabolites were up-regulated and 723 were down-regulated. Moreover, when examining FD against OD, 969 metabolites displayed upregulation while 733 were down-regulated. In the FD versus AD comparison, 886 metabolites were up-regulated and 597 down-regulated. Lastly, in the comparison between AD and OD, 458 metabolites were found to be up-regulated, and 556 were down-regulated (Figure 4B).

#### 3.4.2 GO and KEGG functional annotation analysis of DEPs

**Figure 5:**
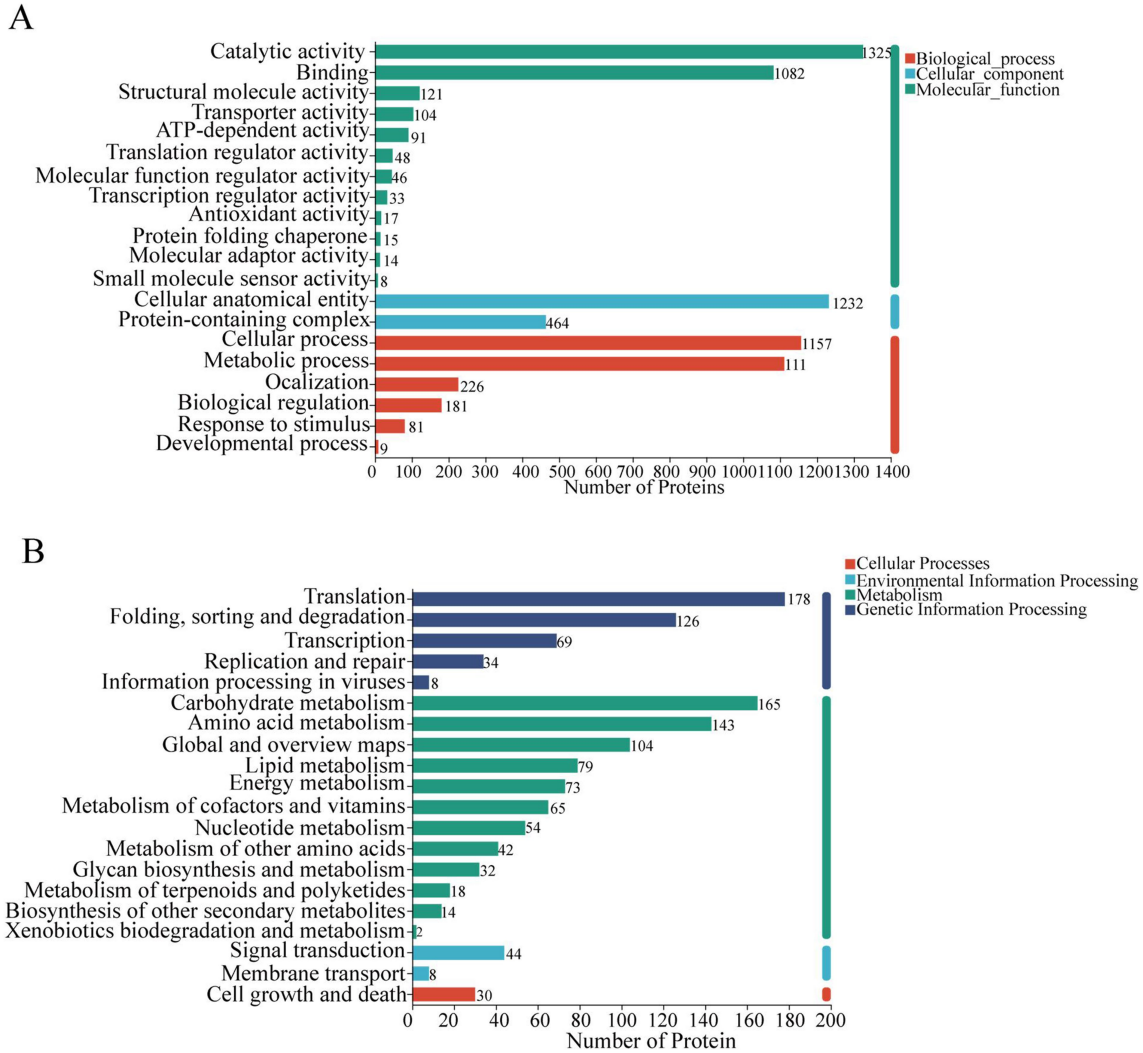
**(A)** Go function annotation results; **(B)** KEGG function annotation results.

To investigate how different drying techniques affect the nutritional and functional properties of *C. sinensis* proteins, we utilized Gene Ontology (GO) analysis and Kyoto Encyclopedia of Genes and Genomes (KEGG) analysis to evaluate the identified DEPs. The functional annotation of GO was applied to all distinct proteins (Figure 5A). An analysis of molecular functions demonstrated that distinct proteins were predominantly enriched in catalytic functions and binding activities. Regarding cellular components, their enrichment was primarily observed in cellular structures and complexes containing proteins. The proteins were predominantly concentrated in cellular process, metabolic process, and localization in relation to biological processes. To gain deeper insight into the biological functions of these proteins, KEGG functional annotation was performed, indicating significant enrichment in metabolism, specifically in pathways pertaining to carbohydrate metabolism, amino acid metabolism, and lipid metabolism (Figure 5B).

#### 3.4.3 KEGG pathway enrichment analysis and KEGG annotation

The KEGG pathway-based analysis enhances comprehension of the biochemical metabolic pathways associated with the identified proteins. To delve deeper into the biological pathways impacted by DEPs from various drying methods, we conducted an enrichment analysis of the identified DEPs using pairwise comparisons of four samples. Different proteins were annotated and enriched using the KEGG pathway database.

**Figure 6:**
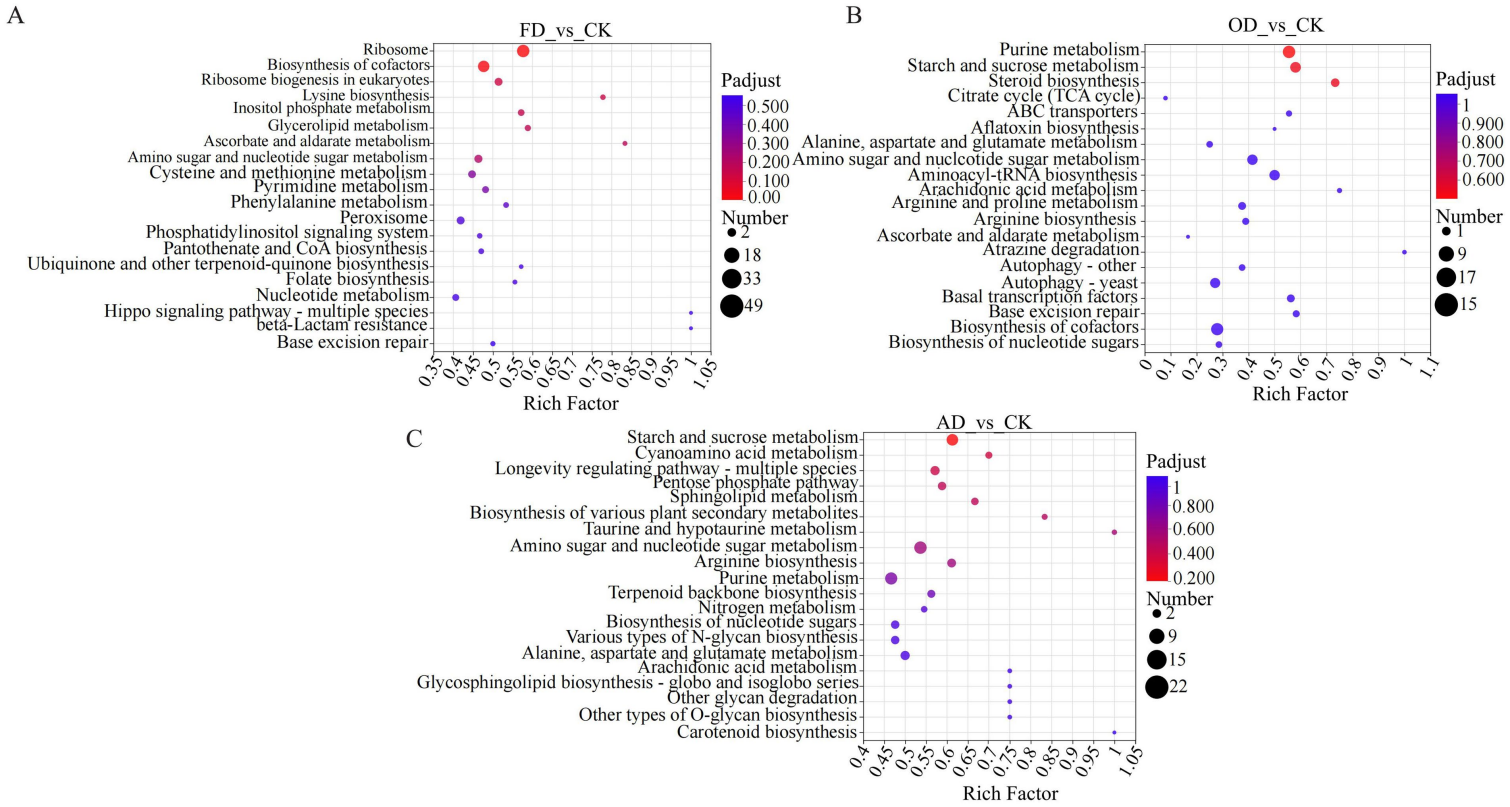
**(A–C)** KEGG enrichment analysis. In the figure, each bubble corresponds to a KEGG pathway. The size of every bubble displays the number of proteins in the protein group that is enriched by the KEGG pathway. The various colors of the bubbles denote the adjusted *p*-value or *p*-value.

The analysis indicated that the proteins expressed differentially in FD compared to CK were significantly enriched in pathways associated with ribosome, cofactor biosynthesis, and eukaryotic ribosome biogenesis. Similarly, the proteins differentially expressed in OD and CK were significantly enriched in pathways related to ribosome, purine metabolism, and nucleocytoplasmic transport. Lastly, the proteins expressed differentially in AD compared to CK were significantly enriched in pathways related to starch and sucrose metabolism, amino sugar and nucleotide sugar metabolism, and purine metabolism. Notably, specific metabolic pathways like Ribosome and Ribosome biogenesis in eukaryotes overlapped and were significantly enriched across all four groups of *C. sinensis* samples. The different proteins in these pathways could potentially act as biomarkers for distinguishing *C. sinensis* under various drying methods. Some of these proteins include serine/threonine-protein kinase rio1 (RIOK1), utp13 domain-containing protein (Utp13), casein kinase II subunit beta, and ribosome biogenesis ATPase rix7 (CKII) (Figures 6A–C).

**Figure 7:**
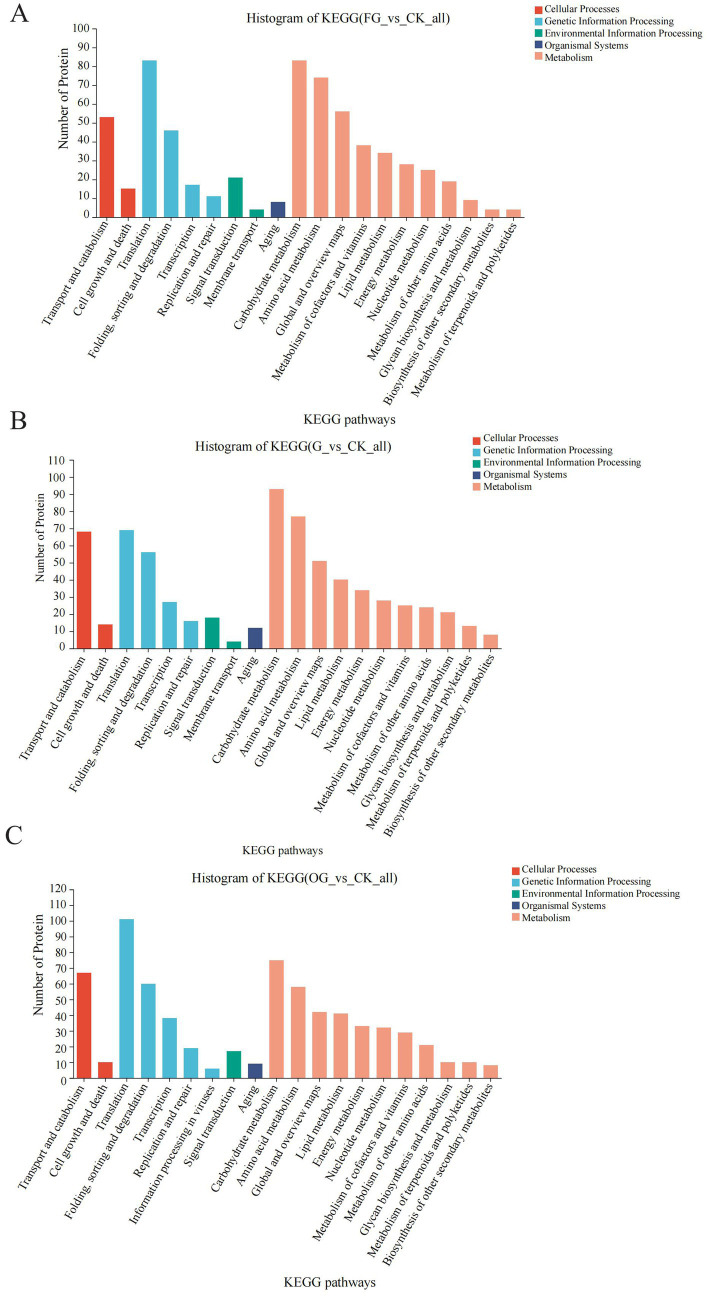
**(A–C)** KEGG function annotation statistics. The KEGG metabolic pathway names are represented on the horizontal axis, while the vertical axis indicates the count of proteins annotated to each pathway.

Considering that *C. sinensis* is regarded as a dietary supplement, it’s critical to comprehend the metabolic alterations linked to the nutrients it contains. In the KEGG annotations, carbohydrate metabolism was a prominent category in the FD vs. CK, AD vs. CK, and OD vs. CK groups, along with amino acid and lipid metabolism. On the other hand, membrane transport is a unique pathway in the FD vs. CK and AD vs. CK groups compared to the OD vs. CK group. Proteins in the membrane transport pathway (multidrug resistance protein 1, etc.) may be key proteins affecting the quality of *C. sinensis*. Local gene regulatory networks and signaling pathways, which are influenced by developmental cues and nutrition sensors, control the metabolism of carbohydrates (33). The results of the KEGG pathway analysis revealed that carbohydrate metabolism is a common enrichment pathway in both the FD vs. CK, AD vs. CK, and OD vs. CK groups. Therefore, the proteins in the carbohydrate metabolic pathway are relatively stable and not easily affected by external conditions, and these proteins are also the main functional proteins in which *C. sinensis* exerts its nutritional value. These results suggest that these critical pathways should be the main focus of research on protein modifications and quality development in *C. sinensis* under various drying conditions (Figures 7A–C).

#### 3.4.4 Subcellular localization of DEPs

**Figure 8:**
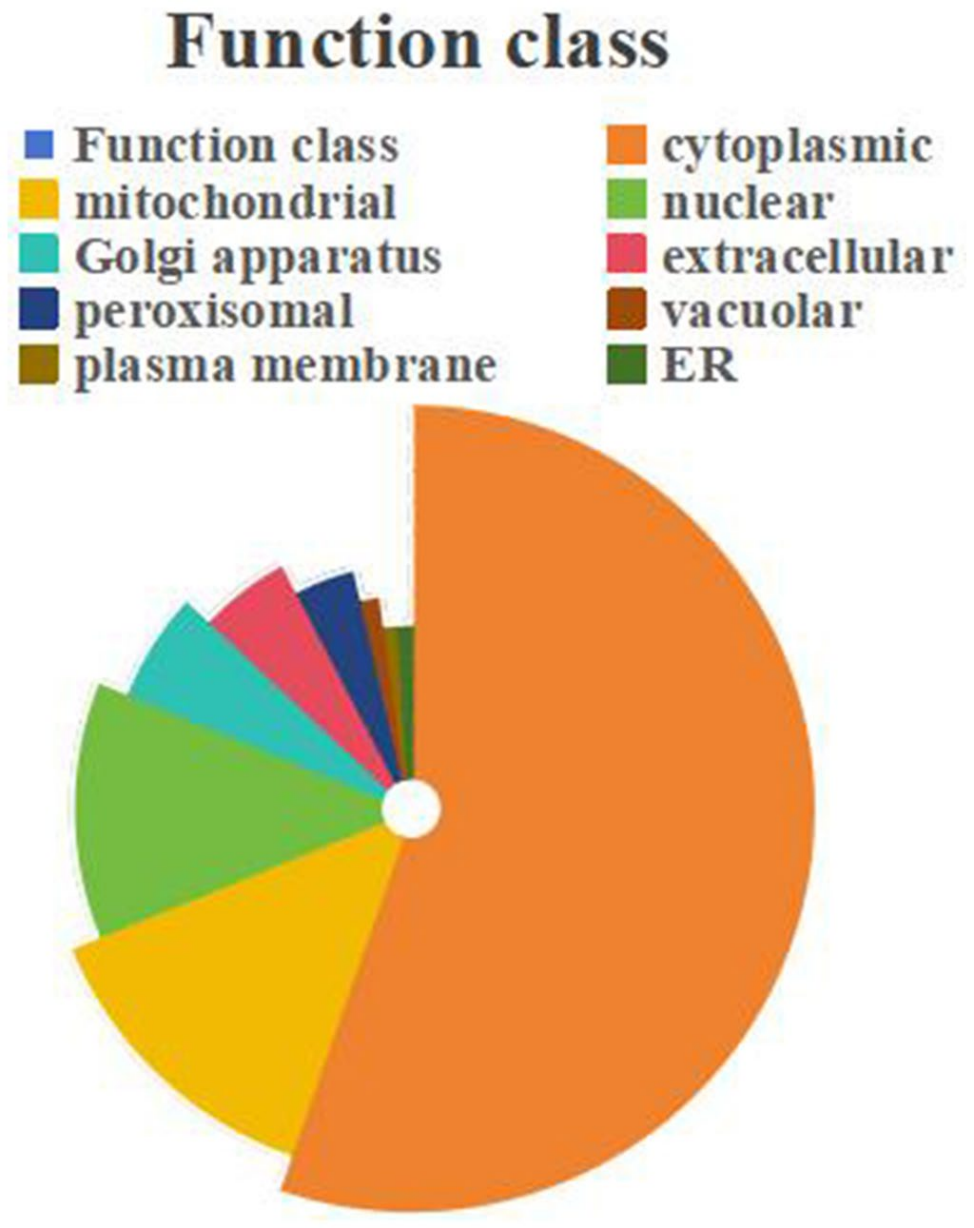
Subcellular localization of DEPs.

The sequence data of all proteins identified in this study were uploaded to the subcellular localization database for comparison through online tools to obtain the subcellular localization information of proteins, which were distributed in cytoplasmic (2015), mitochondrial (491), nuclear (449), and golgi apparatus (221), extracellular (195), peroxisomal (146), vacuolar (52), plasma membrane (43) and er (37). Among them, cytoplasmic accounted for a larger proportion of about 55% (Figure 8).

#### 3.4.5 Protein–protein interaction (PPI) analysis

**Figure 9:**
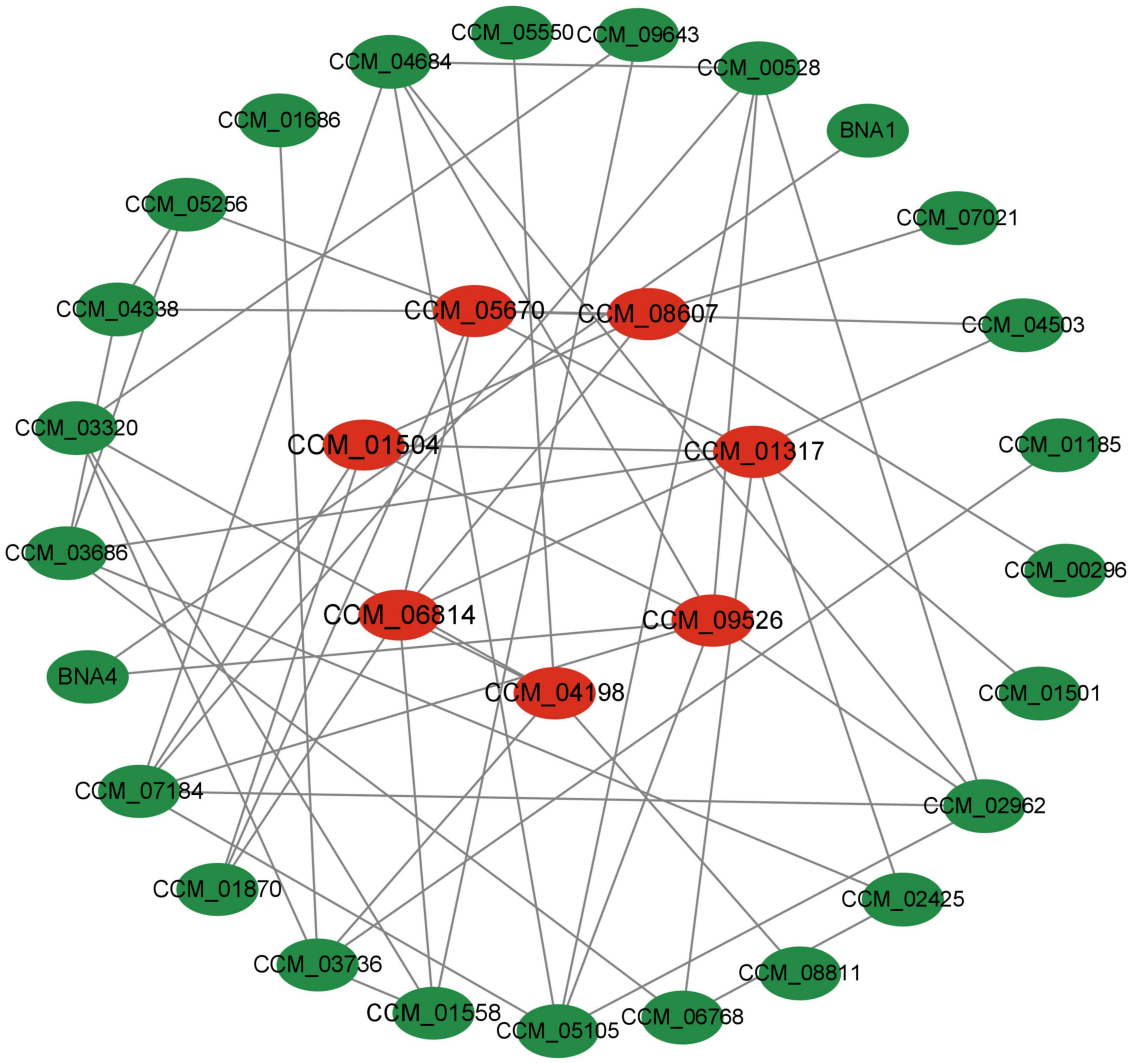
Differential expression protein interaction network [proteins are represented by nodes, while interactions between two proteins are represented by edges. The size of a node is determined by its connectivity (degree), meaning that the more edges connected to a node, the larger it will appear. This signifies the importance of the protein within the network].

Analyzing protein interactions was done for all proteins that showed significant differences. To identify more vital proteins associated with the critical pathway, a protein–protein interaction (PPI) network was established utilizing STRING software with a confidence level exceeding 0.4 (Figure 9). Within the network that was formulated, centrally positioned differentially expressed proteins (DEPs) were identified as pivotal proteins that likely exert significant influence on network regulation. The following proteins were screened based on mediator centrality values such as CCM_05670 (Pantoate-beta-alanine ligase), CCM_08607 (biotin synthase, putative), CCM_01317 (serine hydroxymethyltransferase), CCM_09526 (terpene cyclase), CCM_04198 (glucan phosphorylase), CCM_06814 (6,7-dimethy1-8-ribityllumazine synthase, CCM_01504 (5-aminolevulinate synthase), and others synthase) and other differential proteins significantly interacted with each other. These proteins are more connected and may be key to maintaining the balance and stability of the system.

## 4 Discussion

Prior research has shown that the drying procedure of ancient Chinese herbal remedies is a critical element in assessing their quality, which ultimately impacts their efficacy (34). The drying method is the most commonly employed processing step for *C. sinensis*, making the proteomic differences resulting from various drying techniques a crucial quality factor that warrants comprehensive research for maintaining quality. Both principal component analysis and systematic clustering analysis showed that there were significant changes in CK compared with FD, OD, and AD samples under different drying methods, with OD and AD samples clustered into one group and FD and CK samples clustered into one group with similar protein profiles.

This research compared the protein, content of free sulfhydryl, and disulfide bonds in *C. sinensis* when subjected to various drying techniques against the levels in fresh *C. sinensis*. The drying methods led to a notable reduction in protein and free sulfhydryl levels, while there was a significant rise in disulfide bond content in *C. sinensis* compared to its fresh counterpart. Prior research has indicated that the decline in free sulfhydryl content is linked to the generation of new disulfide bonds. The unfolding of *C. sinensis* proteins under the OD method resulted in the susceptibility of free sulfhydryl groups to oxidation and the formation of inter- or intramolecular disulfide bonds (35). Low temperature promotes the formation of proteins, while high temperature promotes the accumulation of lipids (36). The protein content and disulfide bond levels of the FD sample ranked just below those of the CK sample. This could be due to the reduced levels of oxygen and lower temperature within the vacuum chamber, which help preserve the innate characteristics of *C. sinensis* proteins (14).

A total of 3,639 proteomes were identified in this study, of which 1,190, 1702, 1,483, 1,487, 1,400, and 1,014 proteins were identified as DEPs in the FD vs. CK, FD vs. OD, FD vs. AD, OD vs. CK, AD vs. CK, and AD vs. OD groups. The increase in the number of uniquely abundant proteins in the FD group compared to both the OD and AD groups could be attributed to alterations in the structural and physicochemical characteristics of *C. sinensis* following high-temperature drying, which in turn caused the changes in its functional properties (37). The OD and AD drying methods of *C. sinensis* are both warming processes that carry out a series of organism metabolisms. Both OD and AD drying methods of *C. sinensis* are warming processes and carry out a series of organism metabolism. However, AD warms up gently and the metabolism of the organism is intense, OD warms up rapidly and maintains a temperature above 60°C. High temperature can have an effect on the function of proteins, denatured proteins tend to lose their original biological activity, such as enzyme catalysis is weakened or disappeared. However, the natural air drying is gentle and the biological metabolism in the organism is intense. The significant increase in high-temperature stress experienced results in the generation of a substantial quantity of reactive oxygen species (ROS). These ROS have the potential to harm the proteins’ structure or functionality, resulting in oxidative stress and physiological abnormalities within *C. sinensis* (38, 39). Different drying techniques have a greater effect on the protein of *C. sinensis*, so the commodities on the market of *C. sinensis* under different drying techniques will affect the consumers’ purchasing.

In comparison to the other two drying techniques, samples dried using the FD method exhibited minimal variance in proteins when compared to CK samples. With the FD drying process, *C. sinensis* is surrounded by ice, creating empty spaces within the dry remains as the ice transitions to gas, ultimately maintaining the original composition and function of the product. In addition, proteins tend to deteriorate at high temperatures, and this can greatly reduce or even change the medicinal properties. In contrast, the vacuum freeze-drying process is carried out at low temperatures, and the process breaks down the substance to a small degree (12, 40). GO annotations revealed that the bulk of differential proteins were predominantly concentrated in molecular functions. Meanwhile, KEGG annotations demonstrated that these differential proteins were significantly represented in the Metabolism pathway, with notable enrichment in carbohydrate metabolism and amino acid metabolic pathways. Proteins mainly affected by desiccation include cytokine inducing-glycoprotein, secreted protein, homoserine dehydrogenase and others. Furthermore, the KEGG pathway analysis indicated that *C. sinensis* adapts to drying by modulating its metabolic processes, though these metabolic changes vary considerably with different drying techniques. The choice of drying method significantly influences the proteome of *C. sinensis*, potentially affecting its nutritional and functional properties. The subcellular localization of proteins is a key issue in both molecular and cellular biology and plays a crucial role in proteomics and protein function studies (41). The proportion of cytoplasmic was larger in this study, about 55%. Therefore, the vast majority of proteins in *C. sinensis* are metabolized in the cytoplasm, and the vast majority of chemical reactions take place in the cytoplasmic. Protein is one of the most important components of food, contributing significantly to structural, functional, and sensory properties, which may affect consumer acceptance of processed foods.

Proteins, as the main bearers of life activities, usually need to rely on multiple proteins to mediate and regulate the execution of functions, so the formation of protein interaction networks provides the basis for tapping into the function of proteins (38, 39). Analysis of various differential abundant protein interactions in this study such as CCM_05670 (pantoate-beta-alanine ligase) enzyme class, which has a major impact on cell growth and differentiation, and at the same time participates in the sugar metabolism regulating the intracellular sugar content, provides a new idea for the study of the functional proteins of *C. sinensis* under varying drying techniques.

This research establishes the groundwork for an initial examination of proteomic alterations brought on by the drying process from a proteomics standpoint. Subsequent research ought to concentrate on refining individual drying techniques and delving deeper into function-associated pathways to steer processing procedures and enhance the exploitation of *C. sinensis.* There are some shortcomings in this study, such as possible technical errors and bias in the choice of drying method for protein quantification using only a single histological method.

## 5 Conclusion

Drying is the primary method used to process *C. sinensis*, making the proteomic variances resulting from various drying methods a vital quality concern warranting thorough investigation. In this research, the proteins of fresh *C. sinensis* and samples of *C. sinensis* with different drying methods were quantitatively analyzed by using the 4D-DIA proteomics method. A total of 8,377 proteomes were identified, among which 1,190, 1702, 1,483, 1,487, 1,400, and 1,014 proteins were identified as DEPs for FD vs. CK, FD vs. OD, FD vs. AD, OD vs. CK, AD vs. CK, and AD vs. OD groups, respectively. In addition, both the PCA, and the HCA showed that compared with fresh samples, the proteomes of FD, OD, and AD samples were significantly altered compared to the fresh samples. Compared with fresh *C. sinensis*, the protein content and free sulfhydryl content of dried *C. sinensis* were significantly reduced, and the disulfide bond content was significantly increased. In summary, different drying methods may cause significant changes in the proteome and structure of *C. sinensis*, thus affecting its quality. This study provides theoretical support for further research on the medicinal value of *C. sinensis* on the one hand, and on the other hand, it also provides a brand new idea for the processing and preservation of *C. sinensis*. Other histological methods and other active ingredients will be utilized in subsequent studies to fully exploit its metabolic potential and to address quality issues.

## Data Availability

The original contributions presented in the study are included in the article/Supplementary material, further inquiries can be directed to the corresponding authors.

## Footnotes

^1^ http://string-db.org/

## References

[1] Hc L Hsieh C Lin F. A systematic review of the mysterious caterpillar fungus *Ophiocordyceps sinensis* in Dong Chong Xia Cao (Dong Chong Xia Cao) and related bioactive ingredients. Tradit Complement Med. (2013) 3:16–32. doi: 10.1016/S2225-4110(16)30164-X PMC3924981 24716152

[2] Lu T Zhou L Chu Z Song Y Wang Q Zhao M. *Cordyceps sinensis* relieves non-small cell lung cancer by inhibiting the MAPK pathway. Chin Med. (2024) 19:54. doi: 10.1186/s13020-024-00895-0, PMID: 38528546 PMC10962170

[3] Dai Y Wu C Yuan F Wang Y Huang L Chen Z. Evolutionary biogeography on *Ophiocordyceps sinensis*: an indicator of molecular phylogeny to geochronological and ecological exchanges. Geosci Front. (2020) 11:807–20. doi: 10.1016/j.gsf.2019.09.001

[4] Dong Z Sun X. Chemical components in cultivated *Cordyceps sinensis* and their effects on fibrosis. Chin Herbal Med. (2024) 16:162–7. doi: 10.1016/j.chmed.2022.11.008, PMID: 38375041 PMC10874759

[5] Aung WL Kyaw M. Identification and determination of secondary metabolites and amino acids in Cordyceps. Partners Univ Int Innov J. (2023) 1:251–8. doi: 10.5281/zenodo.8282693

[6] Chiou WF Chang PC Chou CJ Chen CF. Protein constituent contributes to the hypotensive and vasorelaxant activities of *Cordyceps sinensis*. Life Sci. (2000) 66:1369–76. doi: 10.1016/S0024-3205(00)00445-8, PMID: 10755473

[7] Chan RCH Lam SSW Fong FLY Chan DTW Lee FWF Sze ETP. Optimization of protein extraction and two-dimensional gel electrophoresis profiles for the identification of *Cordyceps sinensis* and other similar species. PLoS One. (2018) 13:e0202779. doi: 10.1371/journal.pone.0202779, PMID: 30133529 PMC6105017

[8] Zhang X Liu Q Zhou W Li P Alolga RN Qi L-W. A comparative proteomic characterization and nutritional assessment of naturally-and artificially-cultivated *Cordyceps sinensis*. J Proteome. (2018) 181:24–35. doi: 10.1016/j.jprot.2018.03.029, PMID: 29609095

[9] Tian Y Li D Luo W Zhu Z Li W Qian Z. Rapid freezing using atomized liquid nitrogen spray followed by frozen storage below glass transition temperature for Cordyceps sinensis preservation: quality attributes and storage stability. LWT. (2020) 123:109066. doi: 10.1016/j.lwt.2020.109066

[10] Meng F Wang D. Effects of vacuum freeze drying pretreatment on biomass and biochar properties. Renew Energy. (2020) 155:1–9. doi: 10.1016/j.renene.2020.03.113

[11] Wang J Chen Y Zhao L Zhang Y Fang X. Lipidomics reveals the molecular mechanisms underlying the changes in lipid profiles and lipid oxidation in rape bee pollen dried by different methods. Food Res Int. (2022) 162:112104. doi: 10.1016/j.foodres.2022.112104, PMID: 36461344

[12] Bhatta S Stevanovic Janezic T Ratti C. Freeze-drying of plant-based foods. Foods. (2020) 9:87. doi: 10.3390/foods9010087, PMID: 31941082 PMC7022747

[13] Zan K Zhao L Guo LN Zheng J Ma SC. Comparative study for freeze-drying and sun-drying on multi-index ingredients of *Cordyceps sinensis*. Zhongguo Zhong Yao Za Zhi. (2019) 44:1974–7. doi: 10.19540/j.cnki.cjcmm.20181101.004, PMID: 31355549

[14] Zhang M Xing S Fu C Fang F Liu J Kan J. Effects of drying methods on taste components and flavor characterization of *Cordyceps militaris*. Food Secur. (2022) 11:11. doi: 10.3390/foods11233933, PMID: 36496741 PMC9735880

[15] Bera S Mondal S Rencus-Lazar S Gazit E. Organization of amino acids into layered supramolecular secondary structures. Acc Chem Res. (2018) 51:2187–97. doi: 10.1021/acs.accounts.8b00131, PMID: 30095247 PMC6336168

[16] Fan X Guan G Wang J Jin M Wang L Duan X. Licochalcone a induces cell cycle arrest and apoptosis via suppressing MAPK signaling pathway and the expression of FBXO5 in lung squamous cell cancer. Oncol Rep. (2023) 15:1–14. doi: 10.3389/fphar.2024.1394241, PMID: 37859622 PMC10620845

[17] Wang P-W Hung Y-C Li W-T Yeh C-T Pan T-L. Systematic revelation of the protective effect and mechanism of *Cordyceps sinensis* on diethylnitrosamine-induced rat hepatocellular carcinoma with proteomics. Oncotarget. (2016) 7:60270–89. doi: 10.18632/oncotarget.11201, PMID: 27531890 PMC5312383

[18] Li Q Wu J Yang Q Li H Li F. pH and redox dual-response disulfide bond-functionalized red-emitting gold nanoclusters for monitoring the contamination of organophosphorus pesticides in foods. Anal Chem. (2021) 93:7362–8. doi: 10.1021/acs.analchem.1c01414, PMID: 33961403

[19] Rakita S Pojić M Tomić J Torbica A. Determination of free sulphydryl groups in wheat gluten under the influence of different time and temperature of incubation: method validation. Food Chem. (2014) 150:166–73. doi: 10.1016/j.foodchem.2013.10.128, PMID: 24360434

[20] Tang C-Y Wang J Liu X Chen J-B Liang J Wang T. Medium optimization for high mycelial soluble protein content of *Ophiocordyceps sinensis* using response surface methodology. Front Microbiol. (2022) 13:1055055. doi: 10.3389/fmicb.2022.1055055, PMID: 36569047 PMC9780674

[21] Kumar M Tomar M Potkule J Verma R Punia S Mahapatra A. Advances in the plant protein extraction: mechanism and recommendations. Food Hydrocoll. (2021) 115:106595. doi: 10.1016/j.foodhyd.2021.106595

[22] Lu H Zhou Q He J Jiang Z Peng C Tong R. Recent advances in the development of protein–protein interactions modulators: mechanisms and clinical trials. Signal Transduct Target Ther. (2020) 5:213. doi: 10.1038/s41392-020-00315-3, PMID: 32968059 PMC7511340

[23] Nerbonne JM Kass RS. Molecular physiology of cardiac repolarization. Physiol Rev. (2005) 85:1205–53. doi: 10.1152/physrev.00002.2005, PMID: 16183911

[24] Schöneich C. Thiyl radicals and induction of protein degradation. Free Radic Res. (2016) 50:143–9. doi: 10.3109/10715762.2015.1077385, PMID: 26212409 PMC5118943

[25] Zhang Y Qi P. Determination of free sulfhydryl contents for proteins including monoclonal antibodies by use of SoloVPE. J Pharm Biomed Anal. (2021) 201:114092. doi: 10.1016/j.jpba.2021.114092, PMID: 33984827

[26] Wang C Wang J Zhu D Hu S Kang Z Ma H. Effect of dynamic ultra-high pressure homogenization on the structure and functional properties of whey protein. J Food Sci Technol. (2020) 57:1301–9. doi: 10.1007/s13197-019-04164-z, PMID: 32180626 PMC7054583

[27] Wu X Li F Wu W. Effects of oxidative modification by 13-hydroperoxyoctadecadienoic acid on the structure and functional properties of rice protein. Food Res Int. (2020) 132:109096. doi: 10.1016/j.foodres.2020.109096, PMID: 32331648

[28] Donlao N Wonglek S Bunyameen N Krom WN Chayathatto M Fuggate P. The influence of pretreatments on the quality characteristics and in vitro biological activity of freeze-dried Thai tom-yum ingredients. J Stored Prod Res. (2024) 105:102241. doi: 10.1016/j.jspr.2023.102241

[29] Cui H Yu J Zhai Y Feng L Chen P Hayat K. Formation and fate of Amadori rearrangement products in Maillard reaction. Trends Food Sci Technol. (2021) 115:391–408. doi: 10.1016/j.tifs.2021.06.055

[30] Fu Y Zhang Y Soladoye OP Aluko RE. Maillard reaction products derived from food protein-derived peptides: insights into flavor and bioactivity. Crit Rev Food Sci Nutr. (2020) 60:3429–42. doi: 10.1080/10408398.2019.1691500, PMID: 31738577

[31] Poojary MM Lund MN. Chemical stability of proteins in foods: oxidation and the Maillard reaction. Annu Rev Food Sci Technol. (2022) 13:35–58. doi: 10.1146/annurev-food-052720-104513 34941384

[32] Uddin MP Mamun MA Hossain MA. PCA-based feature reduction for hyperspectral remote sensing image classification. IETE Tech Rev. (2021) 38:377–96. doi: 10.1080/02564602.2020.1740615

[33] Mattila J Hietakangas V. Regulation of carbohydrate energy metabolism in *Drosophila melanogaster*. Genetics. (2017) 207:1231–53. doi: 10.1534/genetics.117.199885, PMID: 29203701 PMC5714444

[34] Wu Q Yan Q Jiang L Chen C Huang X Zhu X. Metabolomics analysis reveals metabolite changes during freeze-drying and oven-drying of *Angelica dahurica*. Sci Rep. (2023) 13:6022. doi: 10.1038/s41598-023-32402-0, PMID: 37055447 PMC10102171

[35] Hu Y Wang L Li Z. Modification of protein structure and dough rheological properties of wheat flour through superheated steam treatment. J Cereal Sci. (2017) 76:222–8. doi: 10.1016/j.jcs.2017.06.013

[36] Zhang Z Gao P Guo L Wang Y She Z Gao M. Elucidating temperature on mixotrophic cultivation of a *Chlorella vulgaris* strain: different carbon source application and enzyme activity revelation. Bioresour Technol. (2020) 314:123721. doi: 10.1016/j.biortech.2020.123721, PMID: 32622276

[37] Zhao K Liu Y Ren Y Li B Li J Wang F. Molecular engineered crown-ether-protein with strong adhesion over a wide temperature range from− 196 to 200 C. Angew Chem. (2022) 134:e202207425. doi: 10.1002/ange.202207425 35726482

[38] Bai X Tan TY Li YX Li Y Chen YF Ma R. The protective effect of *Cordyceps sinensis* extract on cerebral ischemic injury via modulating the mitochondrial respiratory chain and inhibiting the mitochondrial apoptotic pathway. Biomed Pharmacother. (2020) 124:109834. doi: 10.1016/j.biopha.2020.109834, PMID: 31978767

[39] Shen J Zhang D Zhou L Zhang X Liao J Duan Y. Transcriptomic and metabolomic profiling of *Camellia sinensis* L. cv. ‘Suchazao’exposed to temperature stresses reveals modification in protein synthesis and photosynthetic and anthocyanin biosynthetic pathways. Tree Physiol. (2019) 39:1583–99. doi: 10.1093/treephys/tpz059, PMID: 31135909

[40] Zhang L Qiao Y Wang C Liao L Shi D An K. Influence of high hydrostatic pressure pretreatment on properties of vacuum-freeze dried strawberry slices. Food Chem. (2020) 331:127203. doi: 10.1016/j.foodchem.2020.127203, PMID: 32574943

[41] Itzhak DN Tyanova S Cox J Borner GH. Global, quantitative and dynamic mapping of protein subcellular localization. eLife. (2016) 5:e16950. doi: 10.7554/eLife.16950, PMID: 27278775 PMC4959882

